# Enactive-Dynamic Social Cognition and Active Inference

**DOI:** 10.3389/fpsyg.2022.855074

**Published:** 2022-04-29

**Authors:** Inês Hipólito, Thomas van Es

**Affiliations:** ^1^Berlin School of Mind and Brain, Humboldt-Universität zu Berlin, Berlin, Germany; ^2^Department of Psychology, Amsterdam Brain and Cognition, University of Amsterdam, Amsterdam, Netherlands; ^3^Centre for Philosophical Psychology, Universiteit Antwerpen, Antwerp, Belgium

**Keywords:** social cognition, niche construction, active inference, theory of mind, enactivism, dynamical systems theory

## Abstract

This aim of this paper is two-fold: it critically analyses and rejects accounts blending active inference as theory of mind and enactivism; and it advances an enactivist-dynamic understanding of social cognition that is compatible with active inference. While some social cognition theories seemingly take an enactive perspective on social cognition, they explain it as the attribution of mental states to other people, by assuming representational structures, in line with the classic Theory of Mind (ToM). Holding both enactivism and ToM, we argue, entails contradiction and confusion due to two ToM assumptions widely known to be rejected by enactivism: that (1) social cognition reduces to mental representation and (2) social cognition is a hardwired contentful ‘toolkit’ or ‘starter pack’ that fuels the model-like theorising supposed in (1). The paper offers a positive alternative, one that avoids contradictions or confusion. After rejecting ToM-inspired theories of social cognition and clarifying the profile of social cognition under enactivism, that is without assumptions (1) and (2), the last section advances an enactivist-dynamic model of cognition as dynamic, real-time, fluid, contextual social action, where we use the formalisms of dynamical systems theory to explain the origins of socio-cognitive novelty in developmental change and active inference as a tool to demonstrate social understanding as generalised synchronisation.

“In the beginning was the deed.” – Goethe

## Introduction

Because time is continuous and touch and bodily experience form the first interaction with the world, cognition must be embodied. With bodily experience and action, infants first enact the world. They acquire simple motor skills such as walking, reaching, or kicking their legs. These tasks are learnt because infants have some motivation to reach a goal: getting across a room to grab a toy, for example. This motivation forces the exploration of the environment by both bodily experiencing it and learning of patterns: ‘infants come to acquire solutions through exploration: generating movements in various situations and feeling and seeing the consequences of those movements’ (Thelen and Smith, 1996, p. 325; see also Barsalou et al., 2007; Sheya and Smith, 2019). Although the challenge is new when faced with a new task, the cognitive process of moving and perceiving is continuous in time. Through everyday embodied actions, such as poking, squinching, banging and so on, the child gathers understanding about their movements in the environment.

All of this, of course, occurs before language and continues to exist after language. With language and eventually mastering a reasoning toolkit, humans come to conceptually articulate their bodily experience, what they perceive and body action. More precisely, humans can and do use language to describe, think, or picture their bodily experience of the world; even if the phenomenon of linguistic articulation is non-propositional, as it is totally made of bodily experience (Lakoff and Johnson, 2008; Maturana and Varela, 2012; Varela et al., 2016; Hutto and Myin, 2017; Gallagher, 2020).

From this follows that meaning is present even before language. An infant is forced to develop a body skill by first being motivated to reach a meaningful goal, such as reaching a toy or hugging mum and then bodily exploring and thereby experiencing the world. Meaning is here understood in terms of organism-environment attunement. That is to say, *given* the socio-material and historical organism that one is, the habits one has formed and so on, one develops certain sensitivities to the environment it is in. Meaning emerges through these sensitive interactions in the environment. In this sense, ‘meaning’ is not semantic but concerns a relation between organism and environment structured by the interactional history (Varela et al., 2016; Di Paolo et al., 2022).

In the real world, of course, infants and individuals never explore the environment on their own. From as early as birth, learning and understanding the world is social. Family and peers are as much part of the world as the physical objects they interact with. Unlike physical objects, though, humans create an intricate, dynamic network situated and evolving in time. A fundamental aspect of this network is that it is partly held together by meanings. Relationships with family, school and other institutional communities, impart meanings in the same way meaning is made explicit by embodied action. Meaning, as the non-semantic natural attunements, is made explicit by the embodied actions of a specific community. The enculturation with meanings begins with the explorations of the world: as an agent explores, develops and eventually masters a social environment, they become enculturated, where meaning cannot be disentangled from the actions that make meaning explicit. From this standpoint, the understanding of the world then is permeated by meanings, themselves permeating action, thought, imagination and language (Dewey, 1916; Wittgenstein et al., 1969; Hutto et al., 2020).

In cognitive science, social cognition aims to explain how we come to understand these meanings in others and the world. A traditional account is the computational theory of mind (Fodor, 1983; see Sprevak and Colombo, 2018) on the foundations of cognitivism in cognitive science (Haugeland, 1978). Cognitivism is the position counteracting the behaviourist’s *theoria non grata* of the mind as a black box, that cognitive life comes to the computation of mental representations (Pylyshyn, 1980; Dennett, 1982; Fodor, 1983; Sprevak and Colombo, 2018; Smortchkova et al., 2020). Explaining cognition is thereby explaining how information is received, organised, stored and retrieved. For cognitivists, while cognition is a process of developing mental representations about the state of the world, social cognition is a process of developing mental representations of another person’s mental state. The latter is known as mind reading approaches within Theory of Mind (ToM). Pushing against behaviourist and empiricist accounts of newborn’s mind as a ‘blank state’, ToM suggests a hardwired social cognition module that from birth computes mental representations about other people’s beliefs, desires, intentions, emotions, etc. (Scholl and Leslie, 1999; Gerrans, 2002; Stone and Gerrans, 2006; Wellman, 2018; Heyes et al., 2020; Pesch et al., 2020).

Embodied and enactive cognitive science rejects the cognitivist view that understanding others comes down to inferring and attributing mental states in a manner somewhat hardwired from birth. According to the enactivist view, even engagements with the world that involve representational structures, such as thinking, deliberating, or planning, cannot reduce to stored mental objects in the mind of a disembodied spectator. The aim of this paper is two-fold: to dissect new accounts that blend enactivism with inferential accounts and explain why doing so involves a contradiction. The second aim is to offer a reasonable way of linking enactivism and inferential accounts, specifically in the case of social cognition. While some social cognition theories seemingly take an enactive perspective on social cognition, they explain it as the attribution of mental states to other people *via* representational machinery in line with Theory of Mind (ToM). A recent account specifically making this link is Veissière et al.’s (2020) ‘Thinking through other minds’ (TTOM). Holding both enactivism and ToM entails, as we shall see, contradiction and confusion. For ToM holds two assumptions that are widely known to be rejected by enactivism: (1) that social cognition reduces to mental representation and (2) that, at birth, individuals are equipped with an inference toolkit or starter pack for fueling the model-like theorising supposed in (1). The last section advances an enactivist-dynamic model of cognition as dynamic, real-time, fluid, contextual social action. We use the formalisms of dynamical systems theory to explain the origins of socio-cognitive novelty in developmental change and active inference to explain social understanding as generalised synchronisation.

## Thinking Through Other Minds: ‘Enactive’ Inference?

The explanation of neurocognitive processes and psychological experience underlying enculturation aspects of life is a live issue in cognitive science (Colagè and d’Errico, 2020; Enfield and Levinson, 2020; Hutto et al., 2020; Kirmayer et al., 2020). The traditional philosophy of mind and cognitivism, reducing psychological experience to cognitive processes, comprehends cognition in general as a theoretical activity of applying or updating representations. More precisely, as an information-based process that unfolds to the end of computing intelligible representations (Fodor, 1985; Millikan, 2017; Shea, 2018; Piccinini, 2020; Smortchkova et al., 2020; Rupert, 2021). If the mark of the cognitive is representational processes, then, under this account, enculturation is expected to involve representational properties. This reasoning is widely known as ‘Theory of Mind’ (ToM): the capacity to attribute mental states to other people in an accurate way (Scholl and Leslie, 1999; Gerrans, 2002; Stone and Gerrans, 2006; Wellman, 2018; Heyes et al., 2020; Pesch et al., 2020).

Active inference is today a well-known theory of cognition that breaks up with the above computational orthodoxy (Baltieri and Buckley, 2018; Hipólito et al., 2020; Parr et al., 2020) and is increasingly brought to converge with enactivism insights (Constant et al., 2018; Kirchhoff et al., 2018; Korbak, 2021), although this compatibility has been questioned (Di Paolo et al., 2022). Active inference has been employed as a framework for explaining social cognition and the processes of underwriting enculturation (Constant et al., 2018; Gallagher and Allen, 2018; Bolis and Schilbach, 2019; Vasil et al., 2020; Veissière et al., 2020; Hesp et al., 2021; Smith et al., 2022).

Active inference is a modelling theory about how agents act in the environment to maximise their understanding and thereby maintain an appropriate state for their survival and experiential interests. An adaptive system’s action to maximise their adjustment to their local environment can be translated into the constructs of minimisation of uncertainty, entropy, or *surprisal*. As a ‘first principles’ approach to understanding behaviour and the brain, it is framed in terms of a single imperative to minimise free energy given a generative model (Pezzulo et al., 2022). The Free Energy Principle (FEP) states that natural systems remain in non-equilibrium steady states by restricting themselves to a limited number of states. The evolution of systems, that is how a system interacts with the environment, is explained in terms of a free energy gradient at the internal states of the system by variational Bayesian methods (Da Costa et al., 2021). Internal states correspond to an open system’s biomechanical dynamics: a living system (internal states), for example, is situated in an environment (external states).

**Figure 1:**
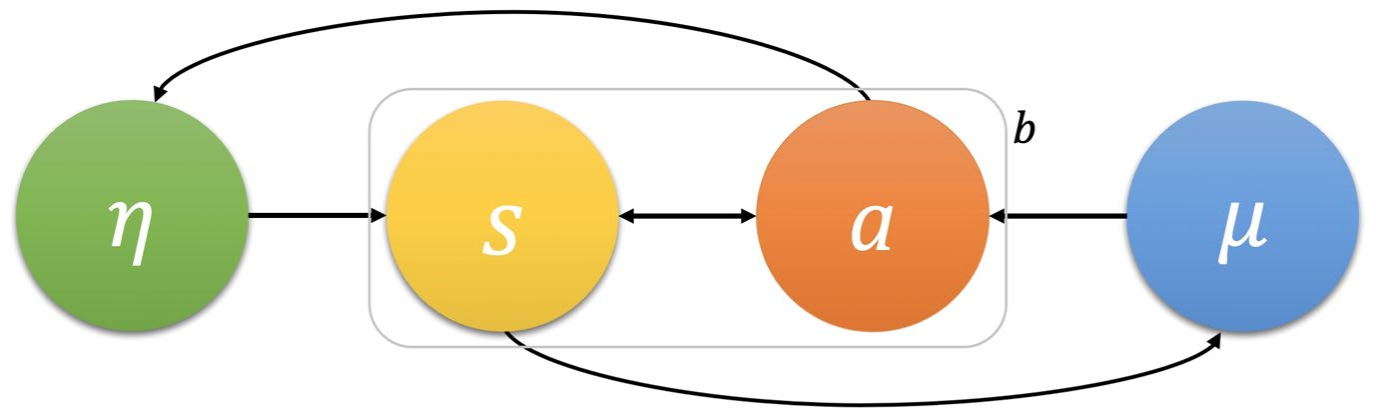
A Markov blanket delineates the conditionally independent internal (*μ*) and external states (*η*) (the arrows represent conditional dependencies between random variables). Considering that there is no arrow between *μ* and *η*, these states are conditionally independent, being indirectly influenced by blanket states (**b**) comprising active (*a*) and sensory (**s**) states. Given its scale-free, this formalism can be applied to explain the influential flows and dynamics of any open system at any scale.

The influences between internal and external states can be highlighted using Markov blankets. A Markov blanket is a scale-free statistical tool that allows us to interpret a natural system’s behaviour as influences between a system and its environment. Because it is a statistical tool of dynamics and flows, it does not necessarily correspond to a physical boundary (e.g., external force in a moving pendulum), even if it sometimes does (e.g., cell exchanging energy in a tissue). A Markov blanket allows for interpreting the activity or behaviour of a system as influences between internal and external states, which indirectly influence one another *via* an additional set of states: *active* and *sensory states*. These states, also directly influencing one another, are called *blanket* states (see Figure 1).

How blanket influence occurs is explained by supposing that internal states of a system engage in an active inference activity: that of predicting the external state. This prediction is made by a generative model, that is a probabilistic model of how external states influence the Markov blanket implicit in internal states’ dynamics.

Here we arrive at an interesting philosophical bifurcation that ties up with the well-known scientific realism debate in philosophy of science. It is possible to understand active inference in two ways: (1) a *realist view* that the properties of the model constructed by applying active inference tools should also be expected to exist as an ontological property in the phenomenon of scientific interest, for example the brain, cognitive, or cell activity and so on. The argument for realism can be made, either by (i) conceiving models as accurate representations (Frege, 1948) or (ii) if they are conceived as abstractions, by their predictive power, if they successfully predict, then the properties of the model should be (literally) present in the system being modelled (Rescorla, 2019), or (iii) claims made about the system, by truth preservation, can also apply to the system, in which case a target system is held to the properties of the model (Dengsø et al., 2022; Kirchhoff et al., 2022; Kiverstein and Kirchhoff, 2022). The second way of thinking of Markov blankets under the philosophical bifurcation is (2) a *non-realist view* that takes the model as simply an instrumental tool that, once applied to some activity, allows the scientist to learn about and draw predictions about a system of scientific interest. However, the system under study does not need to have the properties of the model. Non-realism about Markov blankets can be held in three ways: they are (i) meaning-less (semantic) tools, *viz.* they do not hold semantic value other than that constructed by using them to understand and/or predict behaviour. Following Bas van Fraassen and David Hilbert, scientific theories need not be more than empirically adequate, thus the Markob blanket mathematics is arithmetic and algebraic qualifier-free identities, respectively; (ii) valuable fictions that allow abstracting certain features of a phenomenon (Elgin 2017); (iii) the success of the use of the Markov blanket justifies the acceptance of the epistemic value of the model without necessarily making ontological claims. The success of our best scientific models, for example Markov blanket informed models, does not adequately justify the belief that everything in the model is (approximately) true. In more detail, following Copernicus (1854), hypotheses ‘need not be true nor even probable. And if any causes are devised by the imagination, as indeed very many are, they are not put forward to convince anyone that they are true, but merely to provide a reliable basis for computation’. The hypotheses and assumptions encoded in a computational model are not intended to describe the way the object being modelled is actually structured, but simply served to represent the observed data in an economical fashion (see also, Austin, 1976; Barker and Goldstein, 1998; Borsboom, 2017). A Markov blanket, for example, informs about and represents the reciprocal dynamics between a system and its environment. Elsewhere we have argued that only non-realism about computational models is compatible with enactivism (van Es and Hipólito, 2020) and in this paper we pursue an account of enactive-dynamic social cognition that is in line with (iii).

Veissière et al. (2020) aim to explain the processes underwriting culture acquisition *via* active inference. Taking a realist view on active inference, the authors claim that all aspects of social cognition come down to inference. Departing from an understanding of cognition as embodied and enactive, the authors argue that individuals learn sociocultural shared meanings, habits, norms and expectations by ‘TTOM’: ‘the process of *inferring other agents’ expectations* about the world and how to behave in social context’ by which ‘*information* from and about other people’s expectations constitutes the primary domain of statistical regularities that *humans leverage to predict* and organize behaviour’ (p. 1, emphasis added). Veissière et al.’s (2020) argument for understanding others and the world can be formally put as follows:

**P1**: Social cognition is enactive and embodied.

**P2**: Enactive and embodied the social actor cannot directly grasp social cognition.

**P3**: Anything that cannot be directly grasped must be inferred.

**Conclusion**: Enactive and embodied social cognition must be inferred.

For Veissière et al. (2020), while (P1) cognition is embodied and enactive, because (P2) all aspects and scales of embodied and enactive social understanding are hidden, and (hidden assumption) there is information at all aspects and levels of social engagement, and (P3) what cannot be directly grasped (i.e., requires mediation by a representation) must be inferred, thus (conclusion) no doubt embodied and enactive social cognition must either be or leverage inference.

By P2 and P3 TTOM joins the ToM orthodoxy: understanding the world and others comes down to the ability to infer and attribute mental states (e.g., beliefs, desires, intentions, imagination and emotions; Scholl and Leslie, 1999; Gerrans, 2002; Stone and Gerrans, 2006; Wellman, 2018; Heyes et al., 2020; Pesch et al., 2020):

In helping to solve the puzzle of the *implicit* acquisition of culture, our model provides an integrative view of what has variously been called *mind reading*, perspective taking, joint intentionality, *folk psychology, mentalizing*, or *theory of mind* (TOM) *–* in short, the human ability to ascribe mental states, intentions, and feelings to other human agents and to oneself (Veissière et al., 2020, p. 2, emphasis in the original, although we would highlight the last clause).

In Veissière et al.’s (2020) theoretical model, TTOM, while cognition is understood as embodied and enactive, the social understanding of others is leveraged in mind reading mechanisms under ToM as ‘the process of *inferring other agents’ expectations* about the world and how to behave in social context’ (p. 1). The next section critically assesses TTOM from an enactivist point of view.

## Something’s Gotta Give: Rejecting ‘Enactive’ Inference Through Other Minds

Many well-known philosophical arguments have been raised in recent literature alone by the embodied and enactive cognitive science against the mind reading ToM (Gallagher, 2001, 2006; de Bruin et al., 2011; Hutto et al., 2011; Slors, 2012; Abramova and Slors, 2015; Fernández Castro and Heras-Escribano 2020; Heersmink, 2020; Heras-Escribano, 2020; Hipólito et al., 2020; Lindblom, 2020; see also Menary and Gillett, 2016).

A contradiction between enactivism and ToM is found between P1 and P2 of what we laid out above as Veissière et al.’s (2020) formal argument: social cognition cannot both reduce to inference (P2) AND be embodied/enacted (P1). The contradiction between P1 and P2 results from two hidden assumptions leveraging Veissière et al.’s (2020) argument: (1) that social cognition reduces to mental representation and (2) social cognition is hardwired with an inference toolkit or starter pack for fueling the model-like theorising supposed in (1), which this section critically analyses below. Veissière et al.’s (2020) argument, laid out now with its two hidden assumptions, is constructed as follows:

**P1**: Social cognition is enactive and embodied.

**P2**: Enactive and embodied social cognition cannot directly be grasped by the social actor.

**P3**: What cannot be directly grasped must be inferred.

***Hidden assumption (1)**: social cognition reduces to information in explicit propositional form (mental representation and ascription).*

***Hidden assumption (2)**: social cognition is hardwired with the concepts and logical tools for inference.*

**Conclusion**: Enactive and embodied social cognition must be inferred.

In what follows, we analyse and clarify the contradiction between premises 1 and 2 by critically assessing, from an enactivist perspective, what problems underlie the two assumptions and why enactivists think they should be rejected. It is worth noting that while we critically assess Veissière et al.’s (2020) TTOM formal argument, we take it as a paradigmatic case of ToM. Because TTOM is in perfect alignment with ToM, whatever remarks we make about TTOM will logically apply to ToM, *viz.* any representationalist account of social cognition, or any account of social cognition holding assumptions (1) and/or (2).

### Assumption 1: Social Cognition Reduces to Mental Representation

Enactivism rejects the view that understanding others and the world reduces to mental representation or any form of model-like theorising (Lakoff and Johnson, 2008; Maturana and Varela, 2012; Hutto and Myin, 2013, 2017; Varela et al., 2016; Gallagher, 2020). Because ToM-like theories, on the contrary, defend social cognition as always and everywhere an ascription of a mental and/or neural representation, we find a contradiction between P1 and P2.

Across the board in embodied and enactive cognitive science, no one questions that fully enculturated agents engage in theorising activity with conceptual and reasoning skills. Humans can and do use propositional logic to describe, think, or picture their bodily experience of the world. They write poems essays, measure and map things, paint and draw how they see things from their embodied perspective and offer reasons to explain their actions. The bodily experience of the sociocultural setting is the stuff about which this theorising activity is about.

ToM-like theories suppose, however, that all there is to social cognition is the above form of implicit or explicit theorising. This is so much so that some of the most prominent architects of ToM explain infant development through the analogy between children and scientists: ‘the scientists as a child’ (Gopnik, 1996; Bishop and Downes, 2002). Because the social world is hidden and mysterious from infancy, humans ought to go around developing and testing theories to attain the most plausible explanation of the social everyday world. Social interaction thus exposed delivers a profile of social actors by which they rejected agency: for, under mind reading perspectives, they are not active constructors of a social scene, but instead, on the outside spectators of someone else’s narrative, where much is unknown and thereby requires inferring and adjudicating reasoning *via* the employment of prefabricated models, representations or theories. We reach a contradiction because enactivism widely rejects profiling social actors as passive inferring spectators.

In real-time social interaction, there is little to infer. Social interaction emerges from social actors *co-constructing* a social scene (the scene would not occur without them co-constructing it). Social interaction is replete with non-representational meanings that were there even before social actors were able to speak. An infant is forced to develop a body skill by first being motivated to reach a goal, such as a toy or hugging mum, as something meaningful to them. Meaning has its origins in action, and it is through real-time, fluid, dynamic, contextual action and activity that it is made explicit. From this follows that meanings are present before and regardless of language. It so happens that with mastering a language, humans get to symbolically articulate their bodily, social experiences. In other words, humans get to conceptually articulate experience, that is explain or give reasons for the non-representational stuff they bodily experience in a social scene. But embodied non-representational meanings are regardless of language.

If these embodied meanings are non-representational, what is their profile? They emerge as a co-construction in social action. That is to say, by embodied actions within a specifically enculturated community: for example, how people respond to specific events, how they proceed from one assumption or thought to another, how they organise word afterword, how sentences are said, what reasons they give in favour of an idea, what arguments they raise in what circumstances, what they find interesting and uninteresting and so on. Meanings are not made explicit by language, but that are grasped anyway. They emerge from our engaging in social practices and understanding others without the primacy of explicit theorising, wondering, or inferring. Ultimately, meanings are the links holding sociocultural shared beliefs and stories together: the non-representational aspects involved in social cognition rooted upon a combination of local stories embodied in the individual practices without them being explicitly discussed.

This is so much so that individuals sharing a sociocultural background can see links between the stories that non-enculturated individuals cannot. A cultural clash may result from the failure to see some culturally specific meanings by not having been enculturated in that way, launching them into a ‘spectator’ seat. What the spectator lacks are the enculturated non-explicit meanings. The spectator situation is evident, for example, in ‘second culture’ phenomena (e.g., visiting a new culture, newly ex-pats and refugees; Taguchi, 2019; Ahmed, 2021). Before being specifically enculturated, they experience things from a spectator’s seat. This means that, while they can, in principle, understand the reasons for enculturated practices, the space of reasons does not immediately grant the space of enculturated action.

In this case, the spectator must resort to theoretical activity, that is inference to the best explanation, where this theoretical activity is fully *permeated* by the ways in which the spectator has been otherwise enculturated. Research shows that subjects in this transcultural situation suffer specific mental health issues (Bjertrup et al., 2018; Iandolo and Silva, 2019). Potentially, this lack of meaningful understanding can be partly overcome by community members explicitly offering reasons to the spectator, that is conceptually articulating an explanation of the enculturation meanings the spectator fails to understand (e.g., why someone acted the way they did). But all of this occurs within the space of reasons.

The spectator does not become a (social) actor, *viz.*, does not leave the inference space until they slowly and gradually start enacting these practices themselves. Confronted with a novel sociocultural setting, there is still a form of co-construction, the social actor is there operating in the same space as the locally enculturated people do, and they still participate in some sense in the practices all the while they also infer what is going on. Notably, as agents become enculturated, inference is not necessarily gone, as co-construction is inherently *negotiative*, which can take inferential forms (even if it does not need to). It is in this form that co-constructing the sociocultural niche and forms of life that agents come to be able to provide reasons for why some stories are played out as they are and explain whether they are consistent or conflicting with the enculturated practice. In this respect, Anscombe (2000) remarks that individuals can justify an intention by providing reasons as to why something is done or something would be the case, instead of evidence for why their action or practice is *true*. Truthiness refers exclusively to the inference space of a spectator’s logical reasoning and soundness adjudication, but not to practice. A practice can only be consistent or inconsistent with a cultural picture, where the enactment of a practice reinforces or modifies culturally shared meanings. Notably, culture is enacted and permeates everything that we do, including more intellectual practices such as theorising scientific and philosophical models of the world or parts of it. If understanding others involves enculturated standpoints and practices, social cognition cannot reduce to representational structures with truth-value conditions. While representational structures may be useful when non-explicit meanings fail, that is when someone’s action is ‘alien’ to us, meanings of the enculturated practice (the understandings that are not explicit by language) should take us a long way in our understandings and co-constructions of social scenes. As co-constructors of a social scene, social actors, from a specific enculturated standpoint, non-representational meanings are made explicit, that is, are embodied in everything we do. In doing so, niches are constructed as cultural niches, *viz.* language, rituals, beliefs, tools and so on.

While for ToM-like theories, social cognition comes down to discovering an objective hidden world by means of employing prefabricated, though ever-updating, neurocognitive models; for enactivists, there is no such thing as neurocognitive models: for understanding others takes place at the agent scale, partly allowed by the neural dynamics and processes, naturally. Understanding others is a matter of being skilled at shared non-explicit meanings and potentially some abductive and model-based reasoning,^1^ all of which are context-specific, modifiable and dynamic: here lies an evident contradiction between P1 and P2. The primary issue enactivists take with ToM-like theories is that Tom will not be able to take social actors out of the spectator’s seat. Enactivists do not think that the cultural world is mysterious, nor that culture is the *acquisition* or *transference* of mental objects. The cultural world is not hidden such that it requires understanding through intellectual achievement.

Meanings are out there, made explicit in the actions and permeating everything in between. Meanings cannot be disentangled from the enculturated practices that give rise to them: for one *cannot* simply decide not to be enculturated in a certain way. Even if one can question one’s enculturation structures, shared beliefs and practices, one will do so from our enculturated perspective. This does not mean that non-representational meanings of our experience are not real. They are real, not in the sense of objective reality (whatever this may mean), but they are real experiences standing next to atoms and tables. Indeed, someone interacts the way they do, given the very real, not hidden or mysterious, meanings explicit in the enculturated interaction. From this, culture is not simply *the acquisition* and *transferring* of objects. Culture emerges from communities acting their environments and thereby it is dynamically modifiable: a live museum preserving history but forever reinventing itself by means of member’s actions. This idea has been elaborated under the concept of *intercorporeality* as ‘multimodal’ interaction in complex, material contexts of human life and action in the cultural world (Meyer et al., 2017).

While Veissière et al. (2020) could rejoin our criticism by opting for a two-level approach, according to which basic cognition is enactive and social cognition is inferential, this option would still not be in line with embodied and enactive accounts of cognition. The reason is that social cognition does not consist of a separate subset of cognitive activities. In enactivism, the very idea of social cognition is that cognition as a whole is fundamentally social (Di Paolo et al., 2018). Basic cognition concerns, for example, basic sensorimotor activities, getting and eating food, perceiving and navigating the environment and so on, but these activities are inherently social: our environments are social, populated with others and co-constructively developed by others and ourselves. This means that even the simplest basic cognitive activity is inherently social. The activity always unfolds in relation to the activities (or lack thereof) of others. This means that adopting an inferential account for one type of cognitive activity and not for the other would miss the point by implying that basic and social cognition are two separate sets of activity in the first place.

Understanding and (enculturated) action without leaving the space of reasons will not take the social actor out of the spectator seat: for they will forever sit on the outside making inferences about things instead of acting or enacting. For enactivists, cognition is enacted and embodied, where social action need not but can involve some model-like theorising along with embodied graspings. This abductive reasoning and model-like theorising become more useful as a tool if one lacks the enculturation of a local community, though a space of reasons that does not grant on its own entry in the space of actions, which explains the specific mental health issues emerging in intracultural adjustments.

### Assumption 2: Social Cognition Is Hardwired From Birth

Enactivism rejects the assumption that social cognition is hardwired from birth. Within ToM’s literature, there are two ways of understanding the hardwiring, both of them aligned with the Modularity of Mind, some go as far as to call it Theory of Mind *Module* (ToMM; Gerrans, 2002), that is a computational system that is automatically activated, given ‘social cognition stimuli’, in an encapsulated manner. The first way of understanding the hardwiring is to think that the social cognition-specific module is a fixed mechanism with universal, somewhat nativist properties *a la* Fodor (1983), ‘doomed’ to work in a certain way given a certain stimulus, as Churchland (1996) noted, that is a social cognition full toolkit. The second is a flexible mechanism that revises in the light of new evidence according to hardwired rules (Scholl and Leslie, 1999; Wellman, 2017; Frith, 2019), that is a social cognition ‘starter pack’.

Veissière et al. (2020) do not hold a nativist position. This is made evident given their main goal to determine ‘how culture is *acquired*’. Siding with ToM, they must hold a developmental view of ToM, in which case two challenges are in order. The first is the *circular reasoning* that comes from not spelling out how the starter pack is acquired (note that it is not possible to take a nativist move here). By starter pack, it is meant a flexible system whose models or representations are not universally constrained from birth but can update and upgrade given new evidence and according to hardwired rules, *viz.* Bayesian rules. It is in this sense that the rules are hardwired: they are contained in a generative model with social cognition-specific conceptual machinery allowing for the theorising and adjudicating of mental states to others.

Without a nativist assumption, Veissière et al. (2020) (or any ToM-like theory) need to explain how the starter pack is such that at birth, newborns understand their mother’s face and gestures by means of inference. How can infants intend, move and understand the environment by means of inference without having yet been enculturated with (i.e., having developed) the conceptual machinery for model-based reasoning and adjudication? Without such explanation, their theory is circular:

Newborns acquire culture by inference.

AND

Inference is possible by virtue of being enculturated.

In this setting, the question of how humans become enculturated remains unanswered in the shadows of nativism. What needs to be explained, without a nativist assumption, is the origins of novelty in developmental change. We will answer this question in the next section.

As developing organisms perceive and act in daily life, there must be continuity between these activities and changes over a long-time scale. No one denies the contribution of the nervous system, the hormonal system and the genes (and so on) to non-human animals and human practices and behaviour. But it would be a serious mistake to limit the contributors to those inside the biological system and exclude contributors from outside the organism, such as everyday features of the physical and social environment. Turning things on their head, the question then is how behaviour arises from a multitude of underlying contributing elements. How do these pieces come together as a whole?

ToM-like theories take it that social cognition comes down to inference, where it is not spelt out how individuals come equipped with the tools for inference. This is a problem largely diagnosed by enactivists (Gallagher, 2001, 2006; de Bruin et al., 2011; Hutto et al., 2011; Slors, 2012; Abramova and Slors, 2015; Fernández Castro and Heras-Escribano, 2020; Hipólito et al., 2020; Lindblom, 2020). It is thereby with surprise that we see Veissière et al.’s (2020) TTOM aligning with enactivism, as they say, ‘cognition as an *embodied, enactive*, affective process involving cultural *affordances*’ (p. 1, emphasis added).

In addition, it is also surprising to see Veissière et al.’s (2020) TTOM aligning with Dynamical Systems Theory (DST), a theory well-known for rejecting classic computationalism like mind reading theories. Veissière et al.’s (2020) claim that their TTOM:

Seeks to resolve key debates in current cognitive science, such as… the more fundamental distinction between *dynamical and representational accounts of enactivism*” (p. 1, emphasis added).

But this cannot be the case. DST categorically rejects the notion of representation or cognition as information processing (Favela, 2020). From the classics, we have the insight: ‘rather than computation, cognitive processes may be dynamical systems; rather than computation, cognitive processes may be state-space evolution within these very different kinds of systems’ (Van Gelder, 1995, p. 346).

Seen under the concepts of emergence, non-linearity and change known to be core to DST, social cognition cannot be hardwired from birth, not in the form of representational content (nativist ToM), nor in the form of representational rules (developmental ToM). In DST terms, social cognition processes are not computational but a state-space evolution that is made explicit in the form of the niches constructed by non-human and human communities. From this follows that enculturation processes cannot be conceived of as the *acquisition* and *communication* of static mental objects, but instead as an enactment of the dynamics of a temporally situated social scene. In fact, dynamic theories of social cognition clearly state that:

Our commitment to a biologically consistent theory means that we *categorically reject machine analogies of cognition* and development. The brain may well share certain operations with a digital computer, but it is different from a machine on the most fundamental thermodynamic level… a developmental theory must be appropriate to the organism it serves; thus, *we deliberately eschew the machine vocabulary of processing devices, programs, storage units, schemata, modules, or wiring diagrams.* We substitute, instead, a vocabulary suited to fluid, organic systems, with certain thermodynamic properties” (Thelen and Smith, 1996, p. Xix, emphasis added).

In conclusion, because enactivism categorically rejects any form of hardwired computations, it is in clear contradiction with ToM-like theories. Because DST categorically rejects the analogy between cognition and a computer and machinery vocabulary, ToM-like theories have nothing to offer DST. By the same token Veissière et al.’s (2020) TTOM does not resolve any ‘key debates in current cognitive science, such as… the more fundamental distinction between *dynamical and representational accounts of enactivism’* (p. 1). On the contrary, it brings unnecessary confusion holding upon contradiction.

In what follows, we present an enactivist-dynamic explanation of how we understand others and the world that, while consistent with the description above—of fluid, organic systems, with certain thermodynamic properties—answers questions about the origins of socio-cognitive novelty in developmental change.

## Into the Dynamics of Social Understanding

In the previous section, we have critically assessed the incompatibility between ToM-like theories and enactivism. In doing so we rehearsed and laid out the main features of an enactivist social cognition profile. More precisely, we characterised the activity of understanding others as an activity that is not reducible to mental representations nor hardwired from birth and rejected enculturation as a process of communicating or exchanging goods, *viz.* Mental representational objects. Enculturation instead can be seen as niche construction: the process describing how some living beings, through their activities and choices, modify their own and each other’s niches. A niche refers to the specific natural selection and evolutionary pressures a living being is subject to in its local environment (Odling-Smee et al., 2003; Sterelny, 2012; Laland et al., 2016; Kusano and Kemmelmeier, 2021). Enactivist accounts bring forth a world in three conceptual levels: enaction, niche construction and social construction (Rolla and Figueiredo, 2021).

Dynamical Systems Theory (DST) we shall argue in this section, is an approach that serves to learn about and generate predictive power about the socio-culturally situated practices of agents constructing their niches (Hirsch, 2020; van den Bosch and van der Klauw, 2020). More precisely, we shall explain, under DST, how humans come to develop conceptual toolkits. Importantly, because DST has at its core useful tools such as emergence, non-linearity and spontaneity (etc.) it allows us to move away from prefabricated models of ToM-like theories of social cognition. Despite recent hype, DST is not new. In fact, DST modelling technology, such as network analyses, agent-based modelling, dynamical causal modelling, or differential equations have facilitated some of cognitive science’s most significant early achievements (Favela, 2020) to study diverse cognitive functions (Holmes and Johnsrude, 2020; Barfuss, 2021; Han and Amon, 2021; Tschacher, 2021) and neural activity in neuroimaging studies (Friston et al., 2019).

As a formalism, DST is useful to computationally learn about and understand cognitive behaviour for one major reason: it does not require a realist attitude about the (computational) models used to simulate a behaviour of scientific interest. That is, while DST offers mathematics as well as the computational and mathematical technology to simulate complex behaviour (such as agential practices and behaviour) that would otherwise not be possible to study, it does so without supposing that the agents’ practices or behaviour ontologically entail, involve, or leverage the computational machinery used in the simulation model. This is precisely van Gelder’s insight in his seminal 1995 paper, asking ‘what could cognition be if not computation?’. For him, notably, cognitive processes and agential practices are not computational, but they are the situated observables whose underlying dynamics can be modelled by the DST’s tools. Systems like neuroactivity and behavioural practices are complex system, which means that they are extremely hard to model, or are *intractable* (Garey and Johnson, 1979; Rich et al., 2021). Modelling the dynamics of the system involves too many variables and interrelations for it to be tractable, in other terms, the system has large degrees of freedom, or it lives in high dimensions. How to model on our low dimension computer the high-dimension activity that is generated on a high level is a common problem posed to modelling. A textbook procedure is ‘dimensionality reduction’, *viz.* approximation or optimisation procedure that involves representing in a low dimension, that is a model, some meaningful properties of the data collected from the activity of interest (DeMers and Cottrell, 1993; Beyeler et al., 2019; Tanisaro and Heidemann, 2019). This procedure comes with a cost, a Laplace assumption: that local interacting parts generating behaviour do not interact in a non-linear manner. Although this is useful and insightful, it is simply an instrumental move. That is, a simplification of a complex system into a model that makes complexity tractable. One should therefore refrain from making ontological assumptions, licenced by realism, about the complex system based on the tractable model. Since the tractable model is an opportunity for learning about the complex behaviour, only epistemic claims are allowed. Let us take a closer look of this claim. To make it tractable, we can represent a dynamical system by a static relationship as follows:


(1) yi=f(xi)

With *y* as a dependent variable and *x* as an independent variable, for any possible value of *x1*, a corresponding value will be generated for the dependent variable *y.* In short, the equation describes a static system of a particular value of the variable as a function of the value of another variable or a set of such variables (we critically analyse this below). A static system or model, by definition, will generate predictions without any reference to recursiveness. Making ontological claims from a static model would mean to say that the physical system, just like the model, is static, linear and can be understood as if it were isolated in time and space; and not accounting for its situated and embedded in a dynamical environment. Because we know that the model is reductive (dimensionality reduction) we know we can only make careful, circumscribed epistemic claims with it.

DST takes its instruments as epistemic instruments, not ontological predictors. Understanding cognitive behaviour, including its maturation and enculturation, through a model inherently means to simplify it. Yet this simplification must conserve the system’s characterising features, one of which is *complexity*. An organism situated in its environment is an ensemble of many closely interacting, interdependent components, whose activity is more than the sum of the parts of the components—known as *non-linearity*. Notably, although the situated organism is constantly changing, it maintains coherence over time, that is it is a complex system (Phelan, 2001; Ay et al., 2012; De Domenico et al., 2019). The way the system maintains coherence is explained by the Free Energy Principle (Friston, 2013, 2019; Hipólito, 2019). DST captures the system’s characterising feature: *complexity*. It departs from the observation that things change. Phrased more radically, it makes a key assumption ‘that there is only process’ (Thelen and Smith, 1996, p. 39). As defined by Weisstein (1999): a dynamical model is ‘a means of describing how one state develops into another state over the course of time’, which can be expressed mathematically as:


(2) yt+1=f(yt)

expressing that the next state (at time *t* + 1) is a function, *f*, of the preceding state, at time *t*. In a slightly different notation:


(3) y/t=f(y)

stating that the change of a system, denoted by *y,* over some amount of time, denoted by *t*, is a function *f* of the state of *y*. The function *f* is the dynamical rules specifying some causal principle of change by which the current equation depicts recursive relationships (i.e., *y*t leads to *y*t + 1 and accordingly, *y*t + 1 generates *y*t + 2 and so on).

Applying the DST model in (2) to enculturation aspects of cognition, for example, how a child comes to develop a conceptual kit (since this is not given from birth), that is a child’s growing conceptual toolkit, we obtain the following. The equation describes the current state as a function of a preceding state in a recursive way. This means taking the result of step one in the process (conceptual toolkit today) as the starting value generating the next step (the conceptual toolkit tomorrow). *f* corresponds to the principle of change such that the learning of new concepts at time *t* depends on the concepts already known and the environment the child is situated at (e.g., the people with whom the child communicates at a time *t*; Van Geert, 2009; for recent dynamic approaches to education and learning see Kaplan and Garner, 2020; Koopmans, 2020). This recursiveness illustrates the *enaction*, that is the processes that happen ‘between one behavioural moment and the next’ (Varela, 1992, p. 106; see also Di Paolo et al., 2018, 2022)—which is also characteristic of the dynamical systems approach. An individual’s conceptual toolkit is a niche construction process itself (introduced above): the language we speak, the conversation styles favoured in specific groups, the uses we give to them, how we use them to articulate our and others’ practices, all of this are niche construction practices. Acquiring the abilities to understand and respond to the links between spoken and written patterns, we contribute to niche construction in real time. In languaging, we participate in what constitutes a way of living as a human (Wittgenstein et al., 1969; Hintikka, 1979; Moyal-Sharrock, 2021). After all, ‘we are linguistic/discursive beings and not merely animals with an evolved capacity for language’ (Rouse, 2015, p. 77). This can be frustratingly difficult in our language permeated environment, especially when we find ourselves learning a second language (second language acquisition, or SLA). On the matter, Soleimani (2013) argues that the ‘Newtonian conceptualization of SLA research cannot be comprehensive to deal with the complexities of language acquisition research’ and therefore applies a dynamical systems approach. Languaging is pervasive in that it remains connected to other forms of engagement with the environment: it involves complex non-representational perceptual and practical capacities. For these capacities are present from birth. Because linguistic exchanges are directed, responsive and accountable to our environmental circumstances, language and languaging are better understood as self-organising dynamical systems (Hohenberger, 2011; Di Paolo et al., 2018). In line with this, Elman (1995) explains language not as rule-governed, that is ‘operations on symbols’, but rather embedded in the dynamics of the system permitting movement from certain regions to others, that is navigating the situated environment where languaging happens (see also Patriarca et al., 2020). Importantly, on a DST account, not even languaging is understood in terms of mental representation.

Because languaging is always situated within a wider practical and perceptual context, linguistic capacities are open and incorporate other sensorimotor/cognitive capacities. In this regard, evidence from Nölle et al. (2020) confirms that ‘subtle environmental motivations drive the emergence of different communicative conventions in an otherwise identical task, suggesting that linguistic adaptations are highly sensitive to factors of the shared task environment’. Moreover, the authors speculate that ‘local interactional level, through processes of cultural evolution, contribute to the systematic global variation observed among different languages’ (p. 1). Linguistic articulation, as an enculturated practice, thereby contributes to the material manipulations that further shape the niche we find ourselves in, that is the self-producing process networks of the society, or the long history of niche constructive activities we live in.

Dynamical models offer the opportunity to learn the dynamics of growing conceptual toolkits in languaging: how individuals come to explore and adapt, navigate and socially engage with their environments from one behavioural moment to the next. It does so without reducing this activity to computational neurocognitive processes. By applying dynamical models we learn that it is possible to describe the behaviour generated within the reciprocity between the environment and the organism, such that the specific way that an organism behaves does not exist without the specific way that the environment is and again vice versa.

From an instrumentalist perspective, DST is a useful tool because it seems to capture the right features of self-organising systems. By ‘right features’, we mean the features that require the least philosophical assumptions. Note that, because DST is apt to model self-organising systems, that is offer predictive power, it does not follow that these systems are complex, non-linear, dynamic, etc., in essence. If we do come to ascribe those properties to these systems it must be in virtue of other independent reasons, for example, empirical evidence, not because of DST as a tool for modelling.

## Active Inference in an Enactive-Dynamic Setting

To explain how we understand others, it is necessary to highlight the changes involved in the unfolding enacted and dynamic activity or event in which people participate. Our enactive-dynamic account of cognition posits mental life as an emergence of activities in everyday social life. It provides the biological ground for a cultural and contextual account of how humans understand others and the world. Culture permeates everyday life, where the shared non-representational aspects of that culture permeate how we understand others.

Active inference is an insightful instrument to learn about and understand the dynamics underlying cultural co-construction, considering that it does not necessarily take agents as passive: agents together are the authors of the states of one another (Gallagher and Allen, 2018). Opposed to other process tools, such as prediction error minimisation or predictive processing, placing a boundary of cognition around the skull (Hohwy, 2013) or the skull and the body (Clark, 2015); active inference relies on the Free Energy Principle stating that organisms are coupled with their local environment, *viz.* adjusted, attuned, or synchronised (Friston, 2019; Hipólito, 2019). If agents are coupled with the environment as supposed by active inference, then there is not much in the environment that is hidden from its coupled agents. However, social interaction, under ToM-like theories, such as TTOM, takes the agent as ontologically fully hidden from their environment and social cognition to reduce thereby to inference; it takes the following acyclic description:

Inference of each other’s states → generative models → social action.

**Figure 2:**
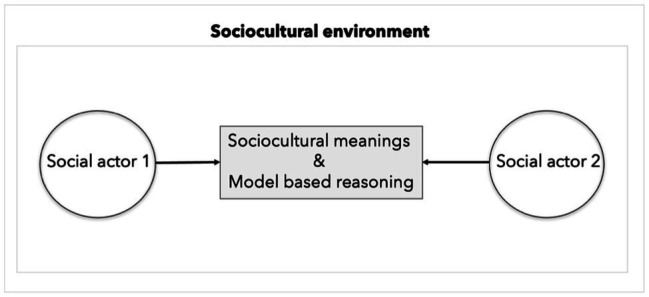
This figure depicts a social interaction between two individuals. Both social actors 1 and 2 understand and contribute further to the construction of sociocultural shared meanings, that is habits and sensitivities to the environment structured by the history of embodied and enactive interactions. In the co-construction of the social scene, because they have been specifically enculturated they employ styles of model-based reasoning specific to the manner they have been enculturated with (e.g., language, social and normative expectations and rituals).

The description above means that, because the world and others are hidden, agents must infer another person’s and the world’s state by means of accurately applying a social cognition generative model. On close inspection, however, we can note that the causality laid out above instead describes that of a scientific practice—*viz.* a scientist whose goal is to understand and predict social dynamics. More precisely, for example, to understand at what point in a conversation a person changes their mind about a certain belief, a modeller first must infer a hypothesis with folk psychology assumptions that will inform and thereby deliver a generative model of how the social scene is expected to unfold knowingly that not every aspect of the social scene can be modeled given it complexity. The likelihood of subject A changing their beliefs about *x*, given the interaction with subject B, in the model would be represented as both agents sharing a generative model or general synchronisation of generative models (Friston and Frith, 2015). But notably, the form of an investigative practice that a scientist engages with is different from that taken by social actors. Because scientists are not social actors, they lack the direct access that social actors otherwise have. This means that if scientists are to understand the dynamics of socialisation and enculturation, they must construct models. Characterising a social scene as analogous to scientific practice is thereby a mischaracterisation. A social scene itself unfolds dynamically as an interactive co-construction by its actors, as shown in Figure 2.

Understanding others and the social world involves the shared sociocultural meanings embedded in practices and some model-based reasoning enabled by the specific ways we have been enculturated with. While understanding others and getting enculturated may sometimes require model-based reasoning, this does not suffice to claim social actors as spectators of their own doing i.e. that all they do is infer. After all they are active actors. They enact the world to adjust it to their existence. Missing this point is missing what active inference is about. Even when engaging in model-based reasoning, this activity is thoroughly permeated by and situated in sociocultural practices and non-representational meanings, for example manners of thinking, shared beliefs and rituals holding a culture.

**Figure 3:**
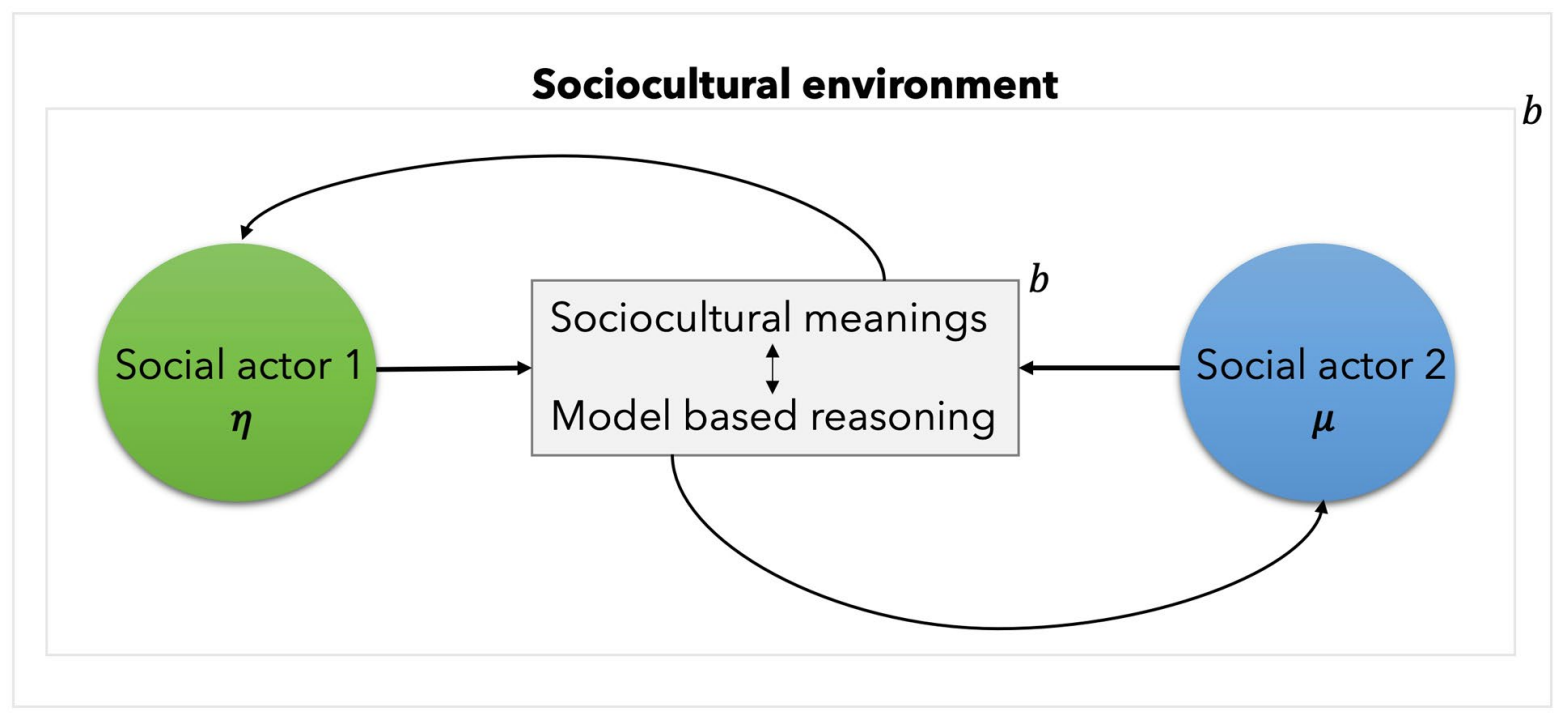
This figure depicts the social action in Figure 2 under the active inference framework. The behaviour of social actor 2 is modelled as internal states coupled with social actor 1 as external states engaging in active inference. The understanding and construction of meanings and the engagement in model-based reasoning are depicted as the balanced influences between sensory and active states or blanket states (**b**). The converse is also possible: it is possible to invert the model such as that social actor 1 becomes internal states and social actor 2 becomes external states.

We can now see how active inference can be useful. Should the scientific question be the finding out of the likelihoods of subject A changing his belief given the interaction with subject B, then an active inference model can be applied, as shown in Figure 3.

Departing from the observation, supported by active inference, that agents are coupled with their local environment, it is possible to model social actors coupled in social interaction. The model must entail a critical assumption: that—for the purposes of the model—social actors *are* (internal) states engaging in belief update or engaging in active inference. The success of using the Markov blanket (***b***) justifies the acceptance of its epistemic value. The epistemic use of the Markov blanket, allows us to learn about and make statements with predictive power about social dynamics if this is on track. But, notably, the profile of the social action does not need to entail ontological claims nor reducing the complexity of (social) phenomena to the mathematical reasoning that we used to scaffold our understanding of it. From this, prediction or inference models are essential tools scientists construct to learn about, understand and predict the patterns in the dynamics of observed behaviour (see Table 1).

**Table 1:** This table distinguishes between **(A)** phenomena observed in the natural world.

A. Natural world	B. Scientific tools/constructs
Synchronised pendulums, neurons, agents Collective intelligence (families, crowds, communities, swarms, flocks of birds)	Dynamical and complex systems theory Active inference Free Energy Principle

In scientific practice, we our reasoning can be guided by principles such as the free energy principle, which inform our construction of models, for example, through the dynamic and complex systems theory, active inference, with the goal of learning more about and be able to predict ***(B)*** observed behaviour in the natural world.

Avoiding ontological claims, we can see that, while there is social understanding, social interaction is not caused by prediction. The observation of social understanding, from social actors co-constructing a social scene, motivates us to scientifically explain it using generative models or in terms of a general *synchronisation of generative models* (Friston and Frith, 2015). But this is not to say that social actors understand each other by simply inferring each other’s states and thereby synchronising models. Note that few would say that pendulums actively infer each other’s states, yet moving pendulums will synchronise their activity at some point (Francke et al., 2020). They behave as if they *knew* each other’s states. They do so, obviously, without following a generative model, *viz.* inferring each other’s states. The reason for this is that it is not prediction/inference that causes the behaviour of pendulums to synchronise. The synchronisation occurs as complex behaviour emerging from the interaction between relatively simple systems (Rihani, 2002; Wolfram, 2018), known as stochastic resonance (Gammaitoni et al., 1998; Lucarini, 2019). Other examples include synchronised neurons, individually interacting as if they *knew* their peer’s behaviour when they could not possibly (Lewis et al., 2004; Friston and Frith, 2015; Balconi et al., 2020; Protachevicz et al., 2021). It is worth noting that, while pendulums do not predict each other’s states, they also do not know each other’s states. At this point, it is important to note a clear distinction between simpler systems like synchronising pendulums and neurons and more complex systems like interacting living beings. Although social interaction does not reduce prediction, agents know much of each other’s behaviour. Much of the meanings unfolding during the construction of the social interaction between enculturated beings is directly grasped in action (e.g., language, social and normative expectations and rituals; Gibson, 2002; Danón and Kalpokas, 2017; Gallagher, 2020).

Active inference is helpful to understand the natural world (Table 1) consisting of phenomena that we understand as complex systems: from synchronised neurons to synchronised social actors. As a corollary of the FEP, active inference provides the mathematical and conceptual tools that can be applied to understand real-world dynamical systems. It can be used in two ways: (1) to build up scientific models of highly complex phenomena. As an information-theoretical function, Variational free energy can be applied to solve for the optimisation of model evidence. This allows for model comparison analysis. Active inference can also be applied to offer insights over (2) the behaviour of self-organising systems. As seen in Figure 3, Markov blankets allow us to interpret the social interaction as the meaningful influences between social actors co-constructing the social environment. In a dynamical setting, they can highlight our patterns of synchronisation (e.g., pendulums or social understanding). In this respect, Friston et al. (2021) have advanced the formalisms that allow us to think of the coupling between internal and external states in terms of generalised synchrony of chaos on the interface between physical and life sciences.

None of the FEP/active inference techniques (1) or (2), however, demand a realist claim for its epistemic virtue. The FEP, as a principle, does not in itself prescribe that complex systems’ behaviour *uses, leverages*, or *are* the models by which the behaviour is scientifically patterned and understood. Models and mathematics are our most prominent scaffolding tools to learn about and predict social behaviour and practices occurring within a complex system. But the system’s complexity shall not be reduced to the behavioural part that we were able to model. If any causal relations are to be devised, they are not to convince anyone of their truthiness, i.e. to describe the way the object being modelled *is*, but merely to serve as the basis for computation, that is to represent the observed data in an economical fashion allowing for learning about the object.

## Conclusion

This paper had a two-fold aim: to dissect new accounts that blend enactivism with inferential accounts and explain why doing so involves a contradiction. We then offered the only reasonable link between enactivism and inferential accounts, that is one that does not include ToM-like assumptions, specifically in the case of social cognition. While some inference models of social cognition seemingly take an enactive perspective on social cognition, they explain it as the attribution of mental states to other people, *via* representational machinery, in line with Theory of Mind (ToM). We have shown that holding both enactivism and ToM entails contradiction and confusion. This is evident when we critically dissect the two hidden assumptions held by ToM-like theories such as TTOM, which are rejected by enactivism: (1) that social cognition reduces to mental representation and (2) that, at birth, individuals are equipped with an inference toolkit or starter pack for fueling the model-like theorising supposed in (1). In our critical assessment, we rehearsed and laid out the main features of an enactivist social-cognitive profile: cognition is enacted and embodied, where social action can involve some model-like theorising if and when embodied understanding is lacking. As co-constructors of a social scene, social actors, from a specific enculturated standpoint, non-representational meanings are made explicit in everything we do. Enaction is the process that happens between one behavioural movement and the next. The formalisms of dynamical systems theory further explain the origins of socio-cognitive novelty in developmental change, and active inference is a suitable, complementary tool to demonstrate the social understanding as generalised synchronisation observed in natural and life sciences.

## Data Availability Statement

The original contributions presented in the study are included in the article/supplementary material, further inquiries can be directed to the corresponding author.

## Author Contributions

All authors listed have made a substantial, direct, and intellectual contribution to the work and approved it for publication.

## Funding

This study was supported by the German Research Foundation (DFG) and the Open Access Publication Fund of Humboldt-Universität zu Berlin.

## Conflict of Interest

The authors declare that the research was conducted in the absence of any commercial or financial relationships that could be construed as a potential conflict of interest.

## Publisher’s Note

All claims expressed in this article are solely those of the authors and do not necessarily represent those of their affiliated organizations, or those of the publisher, the editors and the reviewers. Any product that may be evaluated in this article, or claim that may be made by its manufacturer, is not guaranteed or endorsed by the publisher.

## Footnotes

^1^Model-based reasoning is a theory that attempts to describe the psychological processes that are used when making a logical inference from a given set of premises This can include reason-giving or more sophisticated forms of logics aiming for the development and improvement of psychological models of the world (note that these are not cognitive predictive process models, but models that require aware thinking at the agent level).
